# Contemporary accuracy of death certificates for coding prostate cancer as a cause of death: Is reliance on death certification good enough? A comparison with blinded review by an independent cause of death evaluation committee

**DOI:** 10.1038/bjc.2016.162

**Published:** 2016-06-02

**Authors:** Emma L Turner, Chris Metcalfe, Jenny L Donovan, Sian Noble, Jonathan A C Sterne, J Athene Lane, Eleanor I Walsh, Elizabeth M Hill, Liz Down, Yoav Ben-Shlomo, Steven E Oliver, Simon Evans, Peter Brindle, Naomi J Williams, Laura J Hughes, Charlotte F Davies, Siaw Yein Ng, David E Neal, Freddie C Hamdy, Peter Albertsen, Colette M Reid, Jon Oxley, John McFarlane, Mary C Robinson, Jan Adolfsson, Anthony Zietman, Michael Baum, Anthony Koupparis, Richard M Martin

**Affiliations:** 1 School of Social and Community Medicine, University of Bristol, Canynge Hall, 39 Whatley Road, Bristol BS8 2PS, UK; 2 Department of Health Sciences, University of York and the Hull York Medical School, YO10 5DD, UK; 3 Urology Department, Royal United Hospital, Combe Park, Bath BA1 3NG, UK; 4 Avon Primary Care Research Collaborative, South Plaza, Marlborough Street, Bristol BS1 3NX, UK; 5 School of Social and Community Medicine, University of Bristol, Royal Hallamshire Hospital, Sheffield S10 2JF, UK; 6 Department of Oncology, University of Cambridge, Addenbrooke's Hospital, Box 279 (S4), Cambridge CB2 0QQ, UK; 7 School of Social and Community Medicine, University of Bristol, Freeman Hospital, High Heaton, Newcastle upon Tyne NE7 7DN, UK; 8 Nuffield Department of Surgical Sciences, John Radcliffe Hospital, Oxford OX3 9DU, UK; 9 University of Connecticut Health Center, Farmington, St Francis Hospital and Medical Center, Hartford, CT, USA; 10 Department of Palliative Medicine, Bristol Haematology and Oncology Centre, Bristol BS2 8ED, UK; 11 Department of Cellular Pathology, North Bristol NHS Trust, Southmead Hospital, Bristol BS10 5NB, UK; 12 Department of Cellular Pathology, Royal Victoria Infirmary, Newcastle upon Tyne NE1 4LP, UK; 13 Department of Clinical Science, Karolinska Institutet, Stokholm, Sweden; 14 Harvard Radiation Oncology Program, Harvard Medical School, Massachusetts General Hospital, Boston, MA, USA; 15 Department of Surgery, University College London, Gower Street, London WC1E 6BT, UK; 16 Department of Urology, North Bristol NHS Trust, Southmead Hospital, Bristol BS10 5NB, UK; 17 School of Social and Community Medicine, MRC/University of Bristol Integrative Epidemiology Unit, University of Bristol, Oakfield House, Oakfield Grove, Bristol BS8 2BN, UK

**Keywords:** cluster randomised controlled trial, screening, prostate cancer, prostate cancer mortality, cause of death, death certification, sensitivity, specificity

## Abstract

**Background:**

Accurate cause of death assignment is crucial for prostate cancer epidemiology and trials reporting prostate cancer-specific mortality outcomes.

**Methods:**

We compared death certificate information with independent cause of death evaluation by an expert committee within a prostate cancer trial (2002–2015).

**Results:**

Of 1236 deaths assessed, expert committee evaluation attributed 523 (42%) to prostate cancer, agreeing with death certificate cause of death in 1134 cases (92%, 95% CI: 90%, 93%). The sensitivity of death certificates in identifying prostate cancer deaths as classified by the committee was 91% (95% CI: 89%, 94%); specificity was 92% (95% CI: 90%, 94%). Sensitivity and specificity were lower where death occurred within 1 year of diagnosis, and where there was another primary cancer diagnosis.

**Conclusions:**

UK death certificates accurately identify cause of death in men with prostate cancer, supporting their use in routine statistics. Possible differential misattribution by trial arm supports independent evaluation in randomised trials.

Prostate cancer is the second commonest cause of cancer death in UK men (UK National Screening Committee, 2015). Death certificates are used in routine mortality statistics, large-scale epidemiological studies and randomised controlled trials. However, prostate cancer can be misattributed as the underlying cause of death on death certificates in men diagnosed with prostate cancer (Feuer *et al*, 1999). A review of US medical records (Albertsen *et al*, 2000) suggested that 29% of men with prostate cancer as the underlying cause of death on death certificates had died of some other condition (Albertsen *et al*, 2000).

The possibility of differential attribution bias in trials, where the primary end point is prostate cancer-specific mortality, is also a concern (Black *et al*, 2002). All-cause mortality is least open to bias (Black *et al*, 2002), but because prostate cancer death is relatively uncommon (UK National Screening Committee, 2015), all-cause mortality is less sensitive to the effects of screening.

The possibility of attribution bias has led us and others (Miller *et al*, 2000; de Koning *et al*, 2003; Miller *et al*, 2015) to conclude that assignment of the underlying cause of death in prostate cancer trials must be confirmed by an independent expert (CoDE) committee. We have compared the underlying cause of death determined by an independent CoDE committee, with the underlying cause of death listed on official death certificates in UK men with prostate cancer participating in a UK-wide trial (Lane *et al*, 2014; Turner *et al*, 2014).

## Methods

### Follow-up and identification of a prostate cancer-related event

All 413 000 men enrolled in the Cluster randomised trial of PSA testing for prostate cancer (CAP) trial have been traced and flagged for vital status follow-up at the Health and Social Care Information Centre (Turner *et al*, 2014). Blinded to death certificate and underlying cause of death, detailed information was obtained from the medical records of all men with a potential prostate cancer death (see Supplementary Table 1 for the triggers used to review a potential prostate cancer death), and used to generate a short structured clinical vignette (Williams *et al*, 2015); (see Supplementary Material 1).

### Determination of cause of death

**Figure 1:**
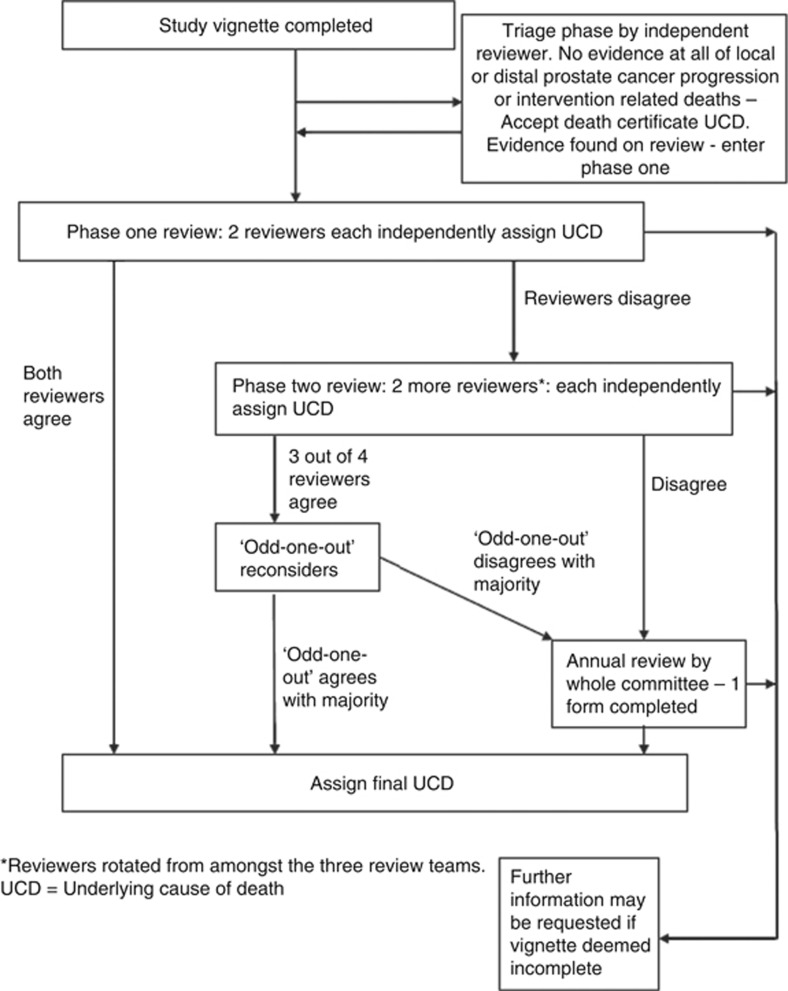
Process for evaluating cause of death.

Members of an international CoDE committee reviewed the vignettes. They completed a questionnaire that when followed in sequence, and using detailed definitions adapted from the European Randomised Study of Screening for Prostate Cancer trial (de Koning *et al*, 2003) acted as an algorithm for assigning cause of death into the following categories: definite, probable, possible, unlikely or definitely not prostate cancer, and definite or probable intervention-related mortality (Supplementary Material 2). The committee was divided into three teams, each comprising four consultants from the following specialties: pathology, palliative care, urology and cancer surgery (see Figure 1 and Supplementary Material 1). The death certificate underlying cause of death was accepted where a review was not triggered (i.e., in the absence of a potential prostate cancer death, these were recorded as due to other causes).

### Analysis

We examined the agreement between prostate cancer assigned as the underlying cause of death on the death certificate with prostate cancer (definite, probable or intervention-related) assigned as the cause of death after expert review. We calculated ‘sensitivity' as the proportion of confirmed prostate cancer deaths as assigned by the expert review process (denominator), which were listed as an underlying cause of death of prostate cancer on the death certificate (numerator) (Box 1). We calculated ‘specificity' as proportion of confirmed non-prostate cancer deaths as above. Each of sensitivity and specificity are accompanied by exact binomial (Clopper–Pearson) confidence intervals (Clopper and Pearson, 1934). We investigated whether sensitivity and specificity varied by age-group (splitting age at death into three groups of approximately equal size), the interval between date of diagnosis and date of death, and the presence of another cancer diagnosis. All parameters were calculated with their respective 95% confidence intervals.

### Ethics

Ethical approval was provided by Trent Research Ethics Committee (MREC/01/4/025; MREC/03/4/093; 05/MRE04/78) and the Confidentiality Advisory Group (PIAG 4–09 (k)/2003; PIAG 1-05(f)/2006).

## Results

Over 50 000 deaths were notified to the CAP investigators by the Health and Social Care Information Centre between 2002 and 2015. Of these, 2069 men had died of a potential prostate cancer-related death, and the underlying cause of death has been established for 1236 (60%) of these men to date.

Table 1 shows the number of deaths assigned as prostate cancer or other cause on the death certificates compared with the expert review process. Of a total of 1236 potential prostate cancer-related deaths, the independent CoDE committee attributed 523 (42%) to prostate cancer and 713 (58%) to other causes. The corresponding cause of death categories based on death certificates were 535 (43%) and 701 (57%), respectively. The expert committee agreed with death certificate derived underlying cause of death (prostate cancer or other) in 1134 cases (92% agreement, 95% CI: 90, 93%).

Eight per cent of deaths categorised as due to other causes after review of the case vignettes had been assigned as prostate cancer on death certificates (death certificate specificity: 92% 95% CI: 90%, 94%). On the other hand, 9% of deaths classified as due to prostate cancer by the reviewers were assigned to other causes on the death certificates (death certificate sensitivity: 91% 95% CI: 89%, 94%).

For men who died within 1 year of their diagnosis of prostate cancer, the death certificates had a sensitivity of 78% (95% CI: 69%, 85%) and specificity of 87% (95% CI: 80%, 93%); for men who died between 1 and 3 years of diagnosis, specificity was 87% (95% CI: 80%, 92%) and sensitivity was 93% (95% CI: 89%, 96%). The presence of another cancer diagnosis, either notified by Health and Social Care Information Centre or present on the death certificate, was also associated with a lower sensitivity (77% 95% CI: 65%, 86%) and specificity (89% 95% CI: 85%, 91%). The age at death had little impact on sensitivity or specificity. Three of the 1236 deaths were categorised as intervention-related deaths by the committee.

## Discussion

These data suggest that relying on underlying cause of death abstracted from official UK death certificates rather than an independent expert committee would result in some misattribution. Specifically, 9% of deaths assigned as being due to prostate cancer by the CoDE committee were recorded on death certificates as deaths from other causes (false negatives), and 8% of deaths considered on the death certificate to be due to prostate cancer were assigned to other causes (false positives) by the expert review. The impact of age was minimal, suggesting the use of UK death certificates could provide a relatively accurate means of evaluating population trends in prostate cancer mortality. However, where there was a death within 1 year of diagnosis of prostate cancer, both false positives (22%) and false negatives (13%) increased. This could reflect a tendency for competing causes of death to be less frequently considered by doctors completing death certificates when a prostate cancer diagnosis has only recently been made. The presence of another primary cancer, either on the death certificate or diagnosed as alive, also resulted in increased false positives (23%) and false negatives (11%). This could be because the clinical picture is unclear in these cases.

These results are based only on those deaths that were triggered for in-depth review by the expert committee because they were potential prostate cancer deaths. We did not review the other 49 000 deaths where there was no evidence of prostate cancer ever being diagnosed or where there was no evidence of other conditions that could have indicated a potential prostate cancer death, such as bone cancer (conceptualised as a potential misclassified bony metastasis). If all these other 49 000 deaths are correctly assumed not to be due to prostate cancer, this will have resulted in near perfect specificity for all deaths.

Similar level of agreement between death certification and expert review were observed in the USA (Albertsen *et al*, 2000), Sweden (Godtman *et al*, 2011) and Finland (Makinen *et al*, 2008). Common reasons for misclassification were cardiovascular or cancer co-morbidities (Albertsen *et al*, 2000). In a recent study (Miller *et al*, 2015), agreement between death certificate and death review committee was >90%, but death certificated causes of death missed treatment-related deaths and the misattribution was differential by trial arm.

The study's strength was that it was based on large trials, we identified intervention-related deaths, and we successfully masked the trial arm from the expert committee (Williams *et al*, 2015), even though this was reported to be difficult in another trial (Barry *et al*, 2013). Limitations are that the results may not be generalisable beyond the cohorts included in the trials, and the assumption that CoDE results were near perfect in accuracy.

UK death certificates provide a relatively accurate means for evaluating cause of death that would be acceptable for routine public health monitoring and large-scale epidemiological studies. In the context of randomised controlled trials, the potential for even a small amount of misattribution bias that is differential by trial arm means that an independent cause of death evaluation is likely to be necessary to provide unbiased outcome data.

## Figures and Tables

**Table 1:** Sensitivity and specificity of prostate cancer as an underlying cause of death on the death certificate *vs* prostate cancer assigned as the underlying cause of death after expert review of clinical vignettes, stratified by age at death, time between diagnosis and death, and presence or absence of another primary cancer diagnosis (*n*=1236)

**Variable**	***N***	**Sensitivity, % (95% CI);** ***N***	**Specificity, % (95% CI);** ***N***
Total	1236	91 (89, 94); *N*=523	92 (90, 94); *N*=713
**Age (years)**			
<65	287	93 (87, 97); *N*=119	92 (86, 95); *N*=168
65–70	365	91 (85, 94); *N*=169	92 (88, 96); *N*=196
>70	584	91 (87, 94); *N*=235	92 (89, 95); *N*=349
**Years between diagnosis and death**			
Not notified via cancer registry	216^a^	100 (54, 100); *N*=6	99 (97–100); *N*=210
<1	231	87 (80, 93); *N*=117	78 (69, 85); *N*=114
1–3	390	93 (89, 96); *N*=249	87 (80, 92); *N*=141
>3	399	92 (87, 96); *N*=151	96 (92, 98); *N*=248
**Other primary cancer present**^b^			
Yes	369	77 (65, 86); *N*=65	89 (85, 92); *N*=304
No	867	93 (91, 96); *N*=458	94 (91, 96); *N*=409

a Sixty-six% were carcinomatosis; also includes PCa on death certificate only.

b In addition to PCa.

## Box 1.

Calculation of sensitivity and specificity

PCa=prostate cancer

CoDE=cause of death evaluation
