# Supplementary material for: Contemporary accuracy of death certificates for coding prostate cancer as a cause of death: Is reliance on death certification good enough? A comparison with blinded review by an independent cause of death evaluation committee
Source: Br J Cancer. 2016 Jun 2;115(1):90–4. doi: 10.1038/bjc.2016.162 (PMC4931376; doi:10.1038/bjc.2016.162)
Supplement: Supplementary Material 1 [file bjc2016162x1.doc]

Supplementary material 1

Methods

***Follow-up and identification of a prostate cancer related event***

The HSCIC supplied the trial investigators with all cancer registrations and information from the death certificates of men who died. Clinical and resource use data were obtained from the medical records of all men with a potential prostate cancer death, defined using the following cause of death algorithm: ICD9 or ICD10 codes for prostate cancer appearing anywhere on the death certificate (part I or II); any death in a man with a diagnosis of prostate cancer or carcinoma in situ of prostate; any man with an ICD9 or ICD10 code on their death certificate for bone cancer (conceptualised as a potential misclassified bony metastasis); or carcinomatosis with an unknown primary. **Supplementary Table 1** presents a full list of ICD9 and ICD10 codes that were reviewed between 2002 until 2012. However, having determined that the ICD9 or ICD10 codes for carcinomatosis with an unknown primary were not identifying any additional deaths classified by the independent cause of death evaluation committee as being prostate cancer deaths, the algorithm was simplified in 2012 to: ‘*a death certificate diagnosis (from an immediate, underlying, or contributing cause-of-death field) that specifies cancer of the prostate (ICD9 185, ICD10 C61), carcinoma in situ of prostate (ICD9 233.4, ICD10 D075); or that suggests a possible misclassified secondary bone cancer (ICD9 170, ICD10 C40, C41), or death from any cause with a previously notified incident prostate cancer.*’

Data items abstracted from the medical records included: symptoms and signs of prostate cancer presence and progression; diagnostic and monitoring tests; histology; tumour stage; treatments received and the outcomes of treatment; complications related to prostate cancer diagnostic investigations or treatments; co-morbidities; and events in the final months before death. These data were extracted by researchers blind to the cancer registration or death certificate information and used to generate a short structured vignette (blinded to trial arm) , which summarised the key clinical events from diagnosis to death. Researchers employed by the study (non clinical background) have been trained on what information to extract from the medical records and how to write the structured vignettes.

***Determination of cause of death***

Cause of Death Evaluation (CoDE) members review the structured vignettes, blinded to trial arm and information on the death certificate (**Figure 1)**. They do not have access to copies of medical records but can request further information if required. An initial triage of the vignettes identified deaths clearly not attributable to prostate cancer or its treatment: i.e. those deaths where there was no evidence of local or distal prostate cancer progression or an intervention related death (**Figure 1**). For the remaining deaths, two reviewers from each team made an initial assessment of the cause of death and if both agreed on whether the death was due to prostate cancer or not, their decision was taken as being the underlying cause of death. If the first two reviewers disagreed, then the vignette was sent to the next two reviewers in the team and a consensus sought based on the views of all four reviewers. In situations where three of the four reviewers agree, there is an opportunity for the reviewer who has assigned cause of death differently, the ‘odd-one-out’, to reconsider their decision in an attempt to reach consensus. If no consensus was reached, then the cause of death was discussed at an annual meeting of the whole CoDE committee (**Figure 1**).

|  | ICD 9 | ICD 10 | Dates |
| --- | --- | --- | --- |
| Malignant neoplasm of prostate* | 185 | C61 | 2002 – present |
| Carcinoma in situ of prostate | 233.4 | D075 | 2002 – present |
| Malignant neoplasm of bone and articular cartilage | 170 | C40, C41 | 2002 – present |
| Malignant neoplasm of male genital organ, site unspecified** | 187.9 | - | 2002 – June 2012 |
| Malignant neoplasm other ill-defined sites, abdomen or pelvis neoplasm, site unspecified** | 195.2, 195.3, 223.9 | - | 2002 – June 2012 |
| Carcinoma in situ of genitourinary system, male genital organs** | 233.6 | - | 2002 – June 2012 |
| Carcinoma in situ of genitourinary system, urinary organs** | 233.9 | - | 2002 – June 2012 |
| Neoplasm of uncertain behavior, prostate** | 236.5 | - | 2002 – June 2012 |
| Neoplasm of uncertain behavior, unspecified male genital organs** | 236.6 | - | 2002 – June 2012 |
| Neoplasm of uncertain behavior, unspecified urinary organs** | 236.9 | - | 2002 – June 2012 |
| Secondary malignant neoplasm of bone & bone marrow** | - | C795 | 2002 – June 2012 |
| Secondary malignant neoplasm of other specified sites** | - | C798 | 2002 – June 2012 |
| Malignant neoplasm of independent (primary) multiple sites** | - | C97 | 2002 – June 2012 |
| Neoplasm of uncertain or unknown behaviour of bone** | - | D480 | 2002 – June 2012 |
| Neoplasm of uncertain or unknown behaviour** | - | D487 | 2002 – June 2012 |
| Neoplasm of uncertain or unknown behaviour, unspecified** | - | D489 | 2002 – June 2012 |
| Neoplasm of uncertain, unknown behaviour, prostate** | - | D400 | 2002 – June 2012 |
| Neoplasm of uncertain, unknown behaviour, other male genital organs** | - | D407 | 2002 – June 2012 |
| Neoplasm of uncertain, unknown behaviour, unspecified male genital organ** | - | D409 | 2002 – June 2012 |
| Neoplasm of uncertain, unknown behaviour, urinary organs** | - | D417 | 2002 – June 2012 |
| Neoplasm of uncertain, unknown behaviour, unspecified urinary organs** | - | D419 | 2002 – June 2012 |
| Sudden death, cause unknown** | 789 | - | 2002 – June 2012 |
| Senility without mention of psychosis** | 797 | - | 2002 – June 2012 |
| Other ill-defined and unknown causes** | 799 | - | 2002 – June 2012 |
| Other sudden death, cause unknown** | - | R96 | 2002 – June 2012 |
| Senility** | - | R54 | 2002 – June 2012 |
| Unknown, unspecified cause of morbidity** | - | R69 | 2002 – June 2012 |
| Carcinomatosis**; where there is no cancer code recorded as underlying cause of death or where one of the cancer codes above is recorded as underlying cause of death, or where incident prostate cancer is notified by HSCIC/cancer registry or carcinomitosis is underlying cause of death (N.B. this was refined (June 2008) to include the caveats listed rather than just the presence of carcinomatosis anywhere on the death certificate.) | 196-199, 239 | C80 | 2002 – January 2012 |
