# Supplementary material for: Contemporary accuracy of death certificates for coding prostate cancer as a cause of death: Is reliance on death certification good enough? A comparison with blinded review by an independent cause of death evaluation committee
Source: Br J Cancer. 2016 Jun 2;115(1):90–4. doi: 10.1038/bjc.2016.162 (PMC4931376; doi:10.1038/bjc.2016.162)
Supplement: Supplementary Material 2 [file bjc2016162x2.doc]

**Supplementary Material 2: CAP Study**

**Cause of Death Evaluation Committee Questionnaire V3.3**

| **ID Number:** | **Review number: Committee** |
| --- | --- |
| **Initials:** |  |
| **DOB:** |  |

**Please complete the following details:**

Committee members: ___________________________________________

_____________________________________________________________

_____________________________________________________________

Date: ___________________________________

**IMPORTANT: Please check the identifiers to ensure that you have the correct questionnaire for the vignette you are reviewing before starting.**

If you have any problems or questions please contact:

Dr Emma Turner

+44 (0)117 9287270

emma.turner@bristol.ac.uk

School of Social and Community Medicine

University of Bristol

Canynge Hall

39 Whatley Road

Bristol

BS8 2PS

UK

**Q0a:** Do the records (including autopsy records if available) support a possible, probable or definite pathologic or clinical diagnosis of prostate cancer?

**Yes** □**_1_**

**No** □**_2_ Go to Q6**

**Q0b:** Was the possible, probable or definite diagnosis of prostate cancer confirmed pathologically?

**Yes** □**_1_**

**No** □**_2_**

**Q0c:** Was the possible, probable or definite prostate cancer clinically present, evident, or active at the time of death or any time during the period leading to death?

**Yes** □**_1_**

**No** □**_2_**

**Uncertain** □**_3_**

**Q0d:** Was the possible, probable or definite prostate cancer metastatic (non-organ confined) at the time of death or any time during the period leading to death?

**Yes** □**_1_**

**No** □**_2_**

**Uncertain** □**_3_**

**Q0e:** Was the man on androgen deprivation therapy for prostate cancer at the time of death or any time during the period leading to death?

**Yes** □**_1_**

**No** □**_2_**

**Uncertain** □**_3_**

**Q0f:** Did the man have evidence of castrate resistant metastatic prostate cancer at the time of death or any time during the period leading to death?

*Castrate resistant implies a rising PSA while the patient is receiving androgen deprivation therapy. Other indicators are initiating chemotherapy or the usual symptoms of advanced prostate cancer that are no longer responding to anti-androgen treatment.*

**Yes** □**_1_**

**No** □**_2_**

**Uncertain** □**_3_**

**Q1a:** Was the death a direct result of metastatic progressing prostate cancer?

(see appendix for definitions)

|  | **Yes (tick one box only)** |
| --- | --- |
| **a) Definite prostate cancer death** | □**_1_ (Go to Q7)** |
| **b) Probable prostate cancer death** | □**_2_ (Go to Q4b)** |
| **c) Possible prostate cancer death** | □**_3_** (Go to Q2) |
| **d) Unlikely prostate cancer death** | □**_4_** (Go to Q2) |
| **e) Definitely not prostate cancer death** | □**_5_** (Go to Q2) |
| **f) Unable to determine** | □**_6_ (Go to Q10)** |
| **Q1b:**  More information needed | □**_1_** (Go to Q1c and END) |

| **Q1c:** If **yes** to Q1b, please describe the additional information you need, and return the form. Additional info will be sent. |  |
| --- | --- |
|  |
|  |

**Q2:** Was the death intervention-related? (tick one box only):

**Definitely** □**_1_ (Go to Q5)**

**Probably** □**_2_ (Go to Q5)**

**Definitely not / Unlikely** (Go to Q3a) □**_3_**

**Q3a:** If **definitely not / unlikely** direct or intervention-related prostate cancer death, could prostate cancer have been a contributory factor?

**Yes** □**_1_** (Go to Q3b)

**No** □**_2_ (Go to Q6)**

**Q3b:** Was the contribution to death the **direct** result of prostate cancer or was the contribution an **indirect** result, arising from associated diagnostic or therapeutic interventions?

**Direct result** □**_1_ (Go to Q6)**

**Indirect result** □**_2_ (Go to Q6)**

**Q4b**: If **probable** prostate cancer death only, what other potential causes of death were there?

**Go to Q7**

**Q5:** If **Definitely** or **Probably intervention-related** death,

|  | **Yes** | **No** |
| --- | --- | --- |
| **Q5a:** Were complications of treatment the cause of death? | □**_1_** | □**_2_** |
| **Q5b:** Were complications of diagnosis / biopsy the cause of death? | □**_1_** | □**_2_** |

**Q5c:** Briefly describe the evidence:

**Go to Q7**

**Q6a:** Was death due to another cancer (not prostate)? That is, did another cancer, or associated medical interventions, initiate or sustain a chain of events leading to death?

**Definitely** □**_1_** (Go to Q6b)

**Probably** □**_2_** (Go to Q6b)

**Possibly** □**_3_** (Go to Q6b)

**Unlikely** □**_4_**  **(Go to Q6c)**

**Definitely not** □**_5_ (Go to Q6c)**

**Q6b:** What was the primary site of the non-prostate cancer?

(State if the death was due to metastatic cancer of unknown origin or you are unsure of the primary)

**Go to Q7**

**Q6c:** Was death due to another non-cancer cause?

**Definitely due to** □**_1_** (Go to Q6d)

**Probably due to** □**_2_** (Go to Q6d)

**Unable to determine** □**_3_**  **(Go to Q7)**

**Q6d:** State the principal cause of death:

(please record only one principal cause of death)

**Q7:** Please rate the quality of the vignette by circling a number on the scale below, where 1 = poor and 10 = excellent

Poor Excellent

1 2 3 4 5 6 7 8 9 10

**Q8:** Please rate your confidence in the cause of death attribution, by circling a number on the scale below, where 1 = not at all confident and 5 = extremely confident

Not at all confident Extremely confident

1 2 3 4 5

|  | **CAP**  Comparison arm | **ProtecT**  Invited for PSA testing arm | **Unsure** |
| --- | --- | --- | --- |
| **Q9a:** What arm of the trial was this man in? | □**_1_** | □**_2_** | □**_3_** |
| **Q9b:** Please state reasons for decision: |  | | |
|  |  | | |
| **Q9c:**  If you answered ‘**ProtecT**’ to 9a, what primary treatment arm was the man assigned to? | **Active Monitoring** □**_1_**  **Radiotherapy** □**_2_**  **Radical prostatectomy** □**_3_**  **Non-responder to invite** □**_4_** | | |
| **Q9d:** Please state reasons for decision: |  | | |
|  |  | | |

| **Q10a:** This committee is unable to determine if prostate cancer death  **Q10b:** If so, please give details (continue on reverse if necessary): | □ |
| --- | --- |
|  |  |
